# Supplementary material for: Siraitia grosvenorii Extract Attenuates Airway Inflammation in a Mouse Model of Respiratory Disease Induced by Particulate Matter 10 Plus Diesel Exhaust Particles
Source: Nutrients. 2023 Sep 25;15(19):4140. doi: 10.3390/nu15194140 (PMC10574535; doi:10.3390/nu15194140)

Supplementary data

Effects of Siraitia grosvenorii Extract (SGE) on PM10-induced MAPK/NF-κB signaling in lung tissue.

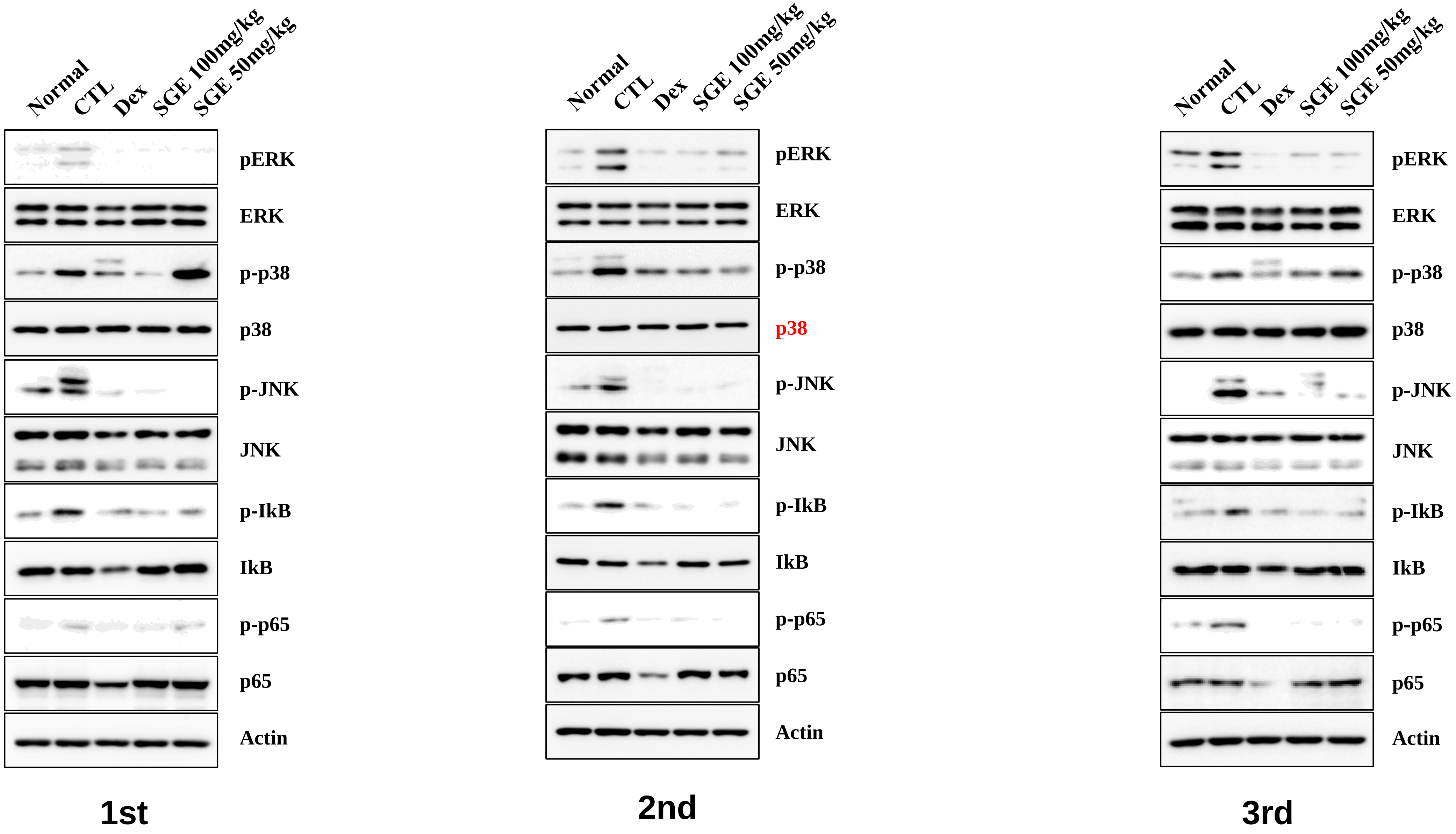

Effects of Siraitia grosvenorii Extract (SGE) on PM10-induced MAPK/NF-κB signaling in lung tissue.

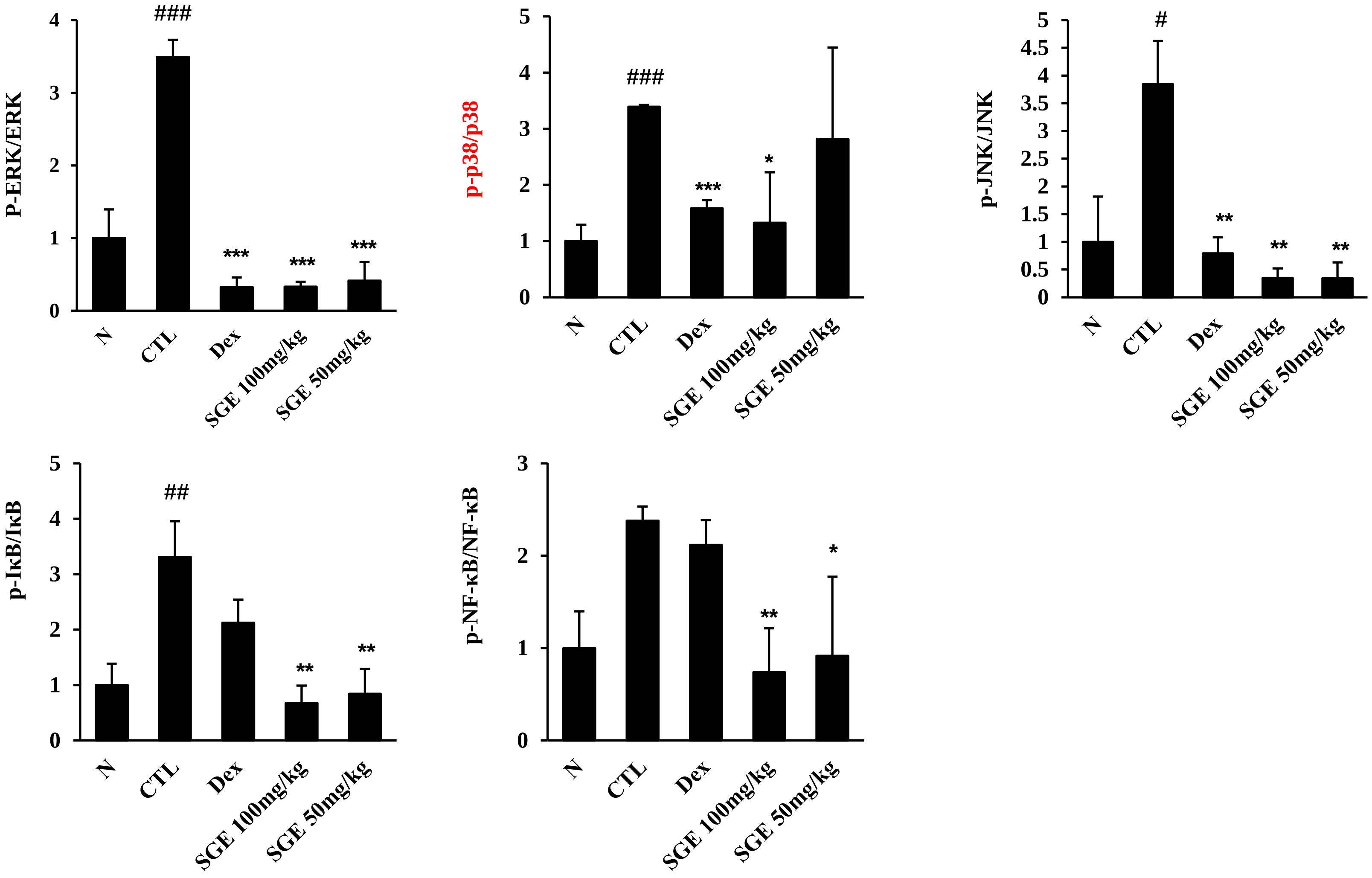

PM10 induced lung inflammation_lung tissue WB_1

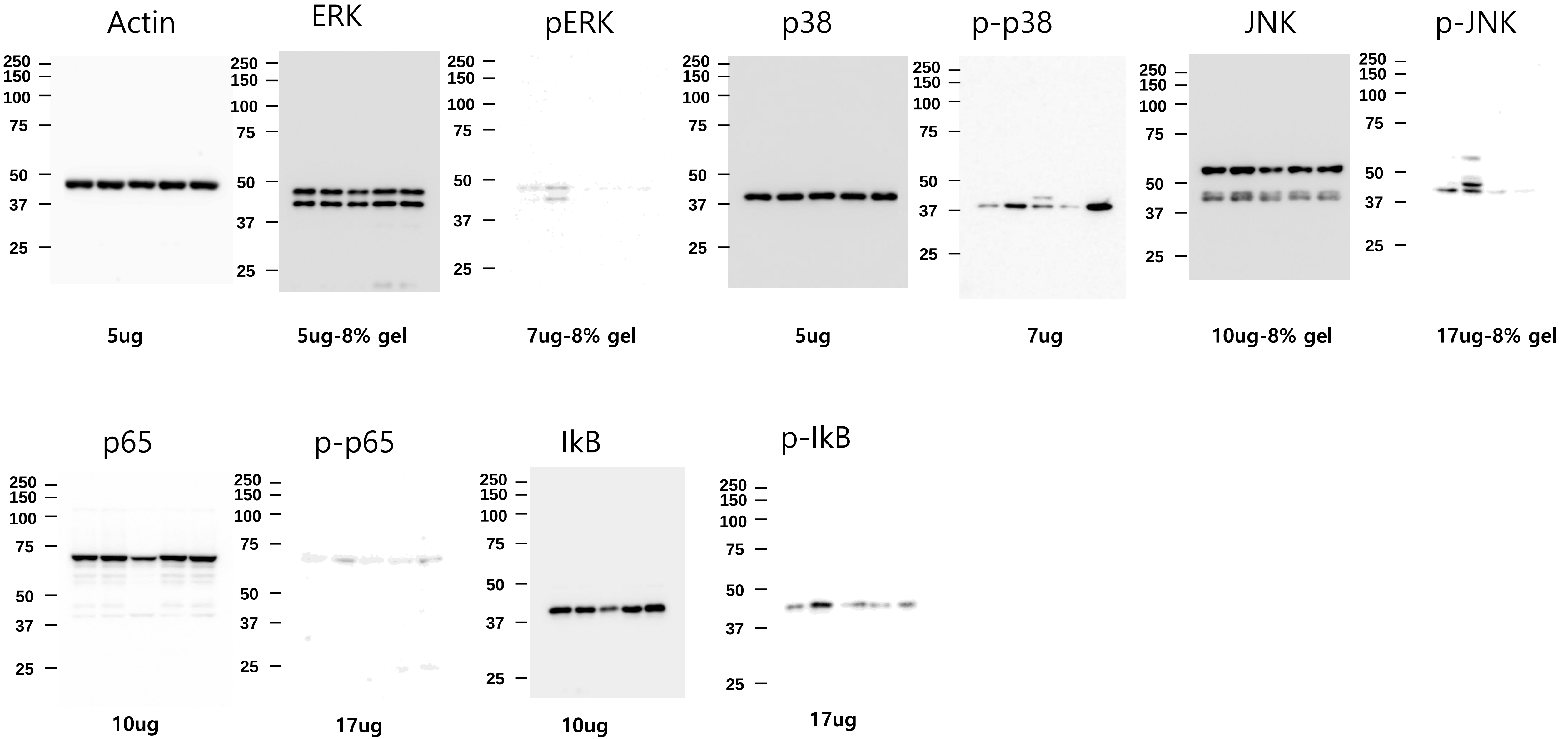

PM10 induced lung inflammation_lung tissue WB_1

ERK mem -> Actin

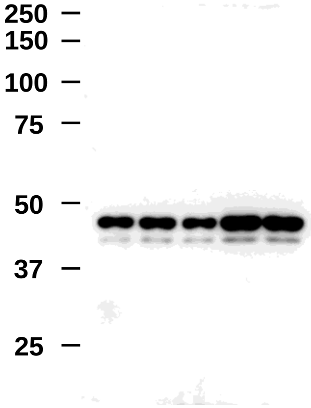

pERK mem -> Actin

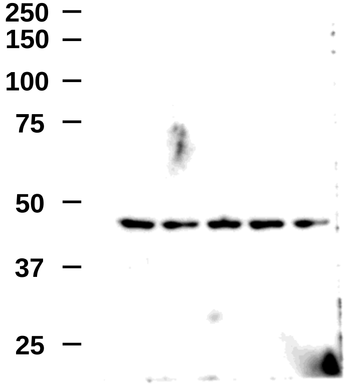

p38 mem -> Actin

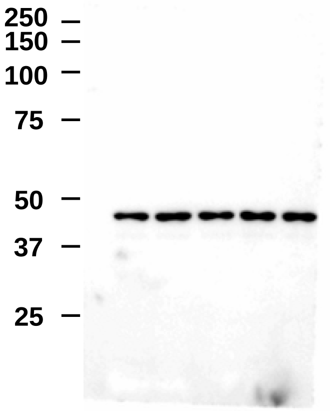

p-p38 mem -> Actin

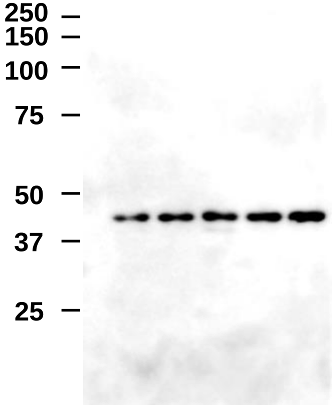

JNK mem -> Actin

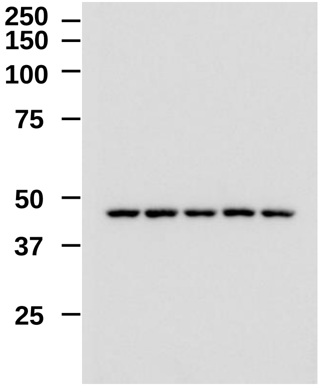

p-JNK mem -> Actin

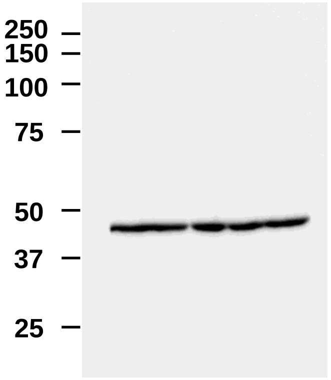

p65 mem -> Actin

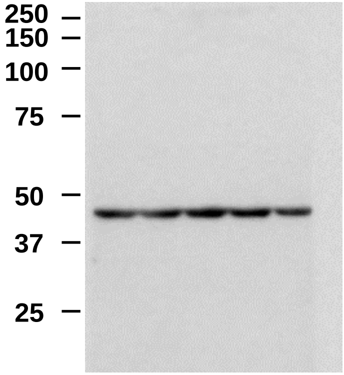

p-p65 mem -> Actin

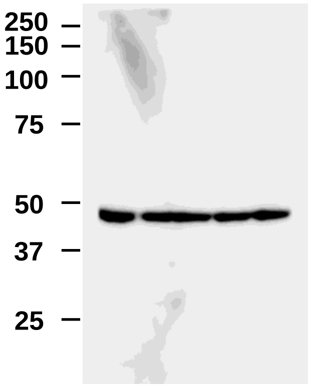

IkB mem -> Actin

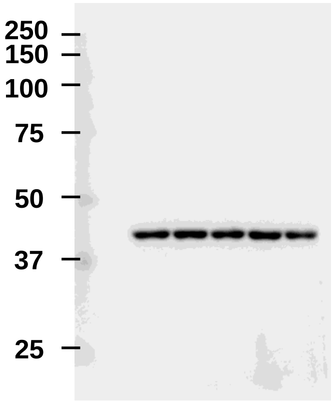

pIkB mem -> Actin

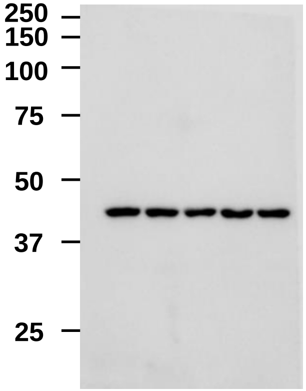

PM10 induced lung inflammation_lung tissue WB_2

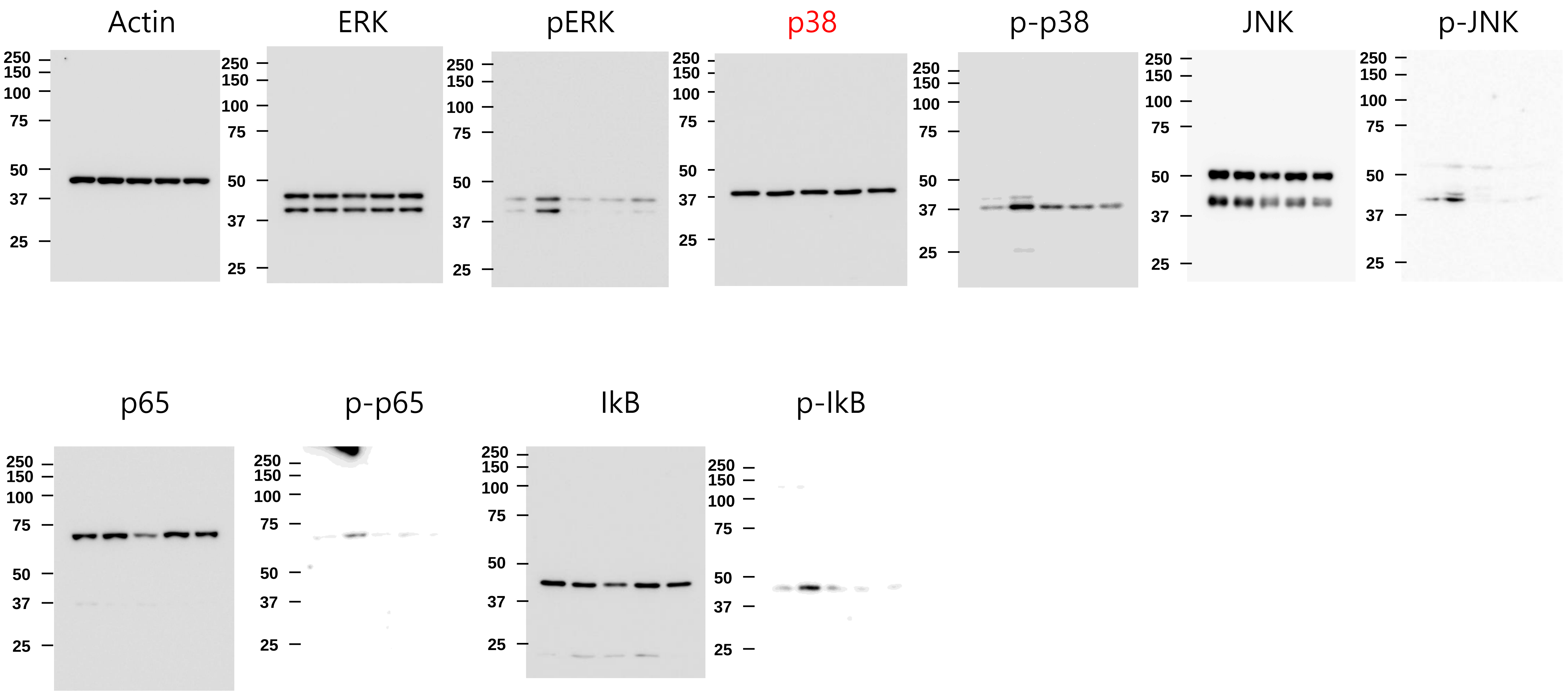

PM10 induced lung inflammation_lung tissue WB_2

ERK mem -> Actin

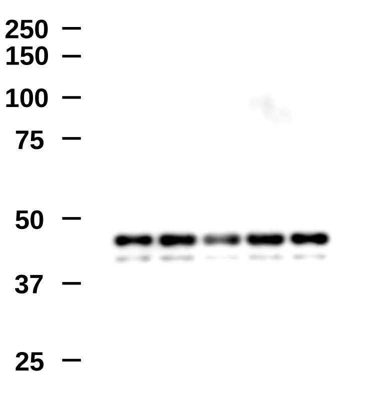

pERK mem -> Actin

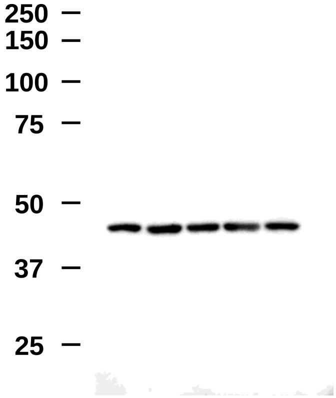

p38 mem -> Actin

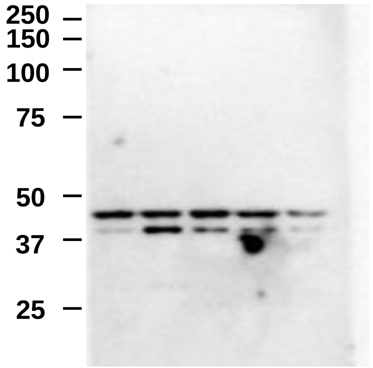

p-p38 mem -> Actin

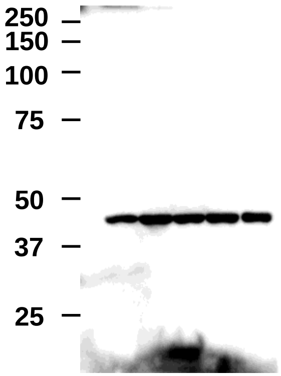

JNK mem -> Actin

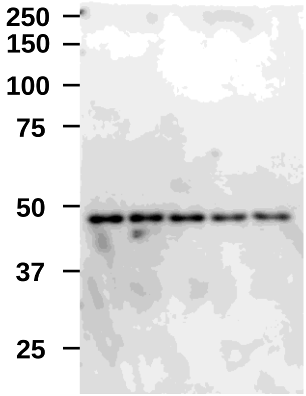

p-JNK mem -> Actin

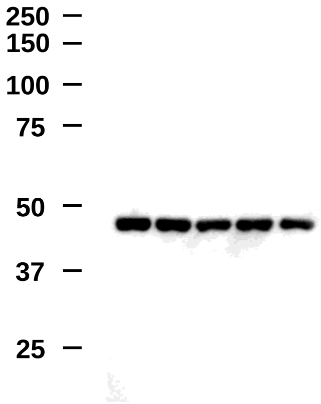

p65 mem -> Actin

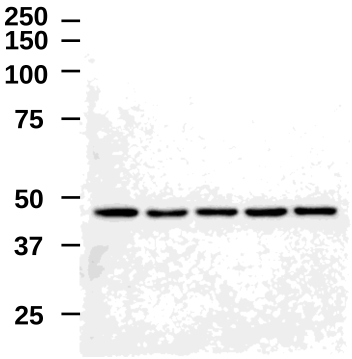

p-p65 mem -> Actin

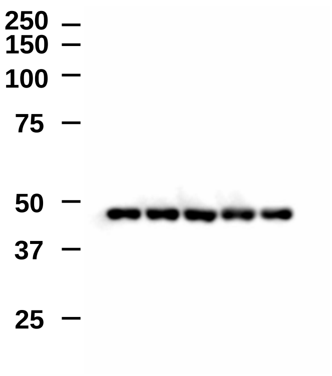

IkB mem -> Actin

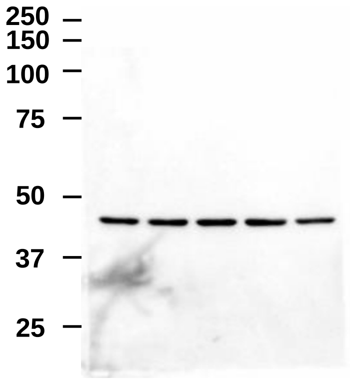

pIkB mem -> Actin

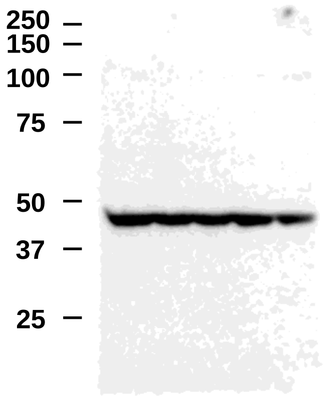

PM10 induced lung inflammation_lung tissue WB_3

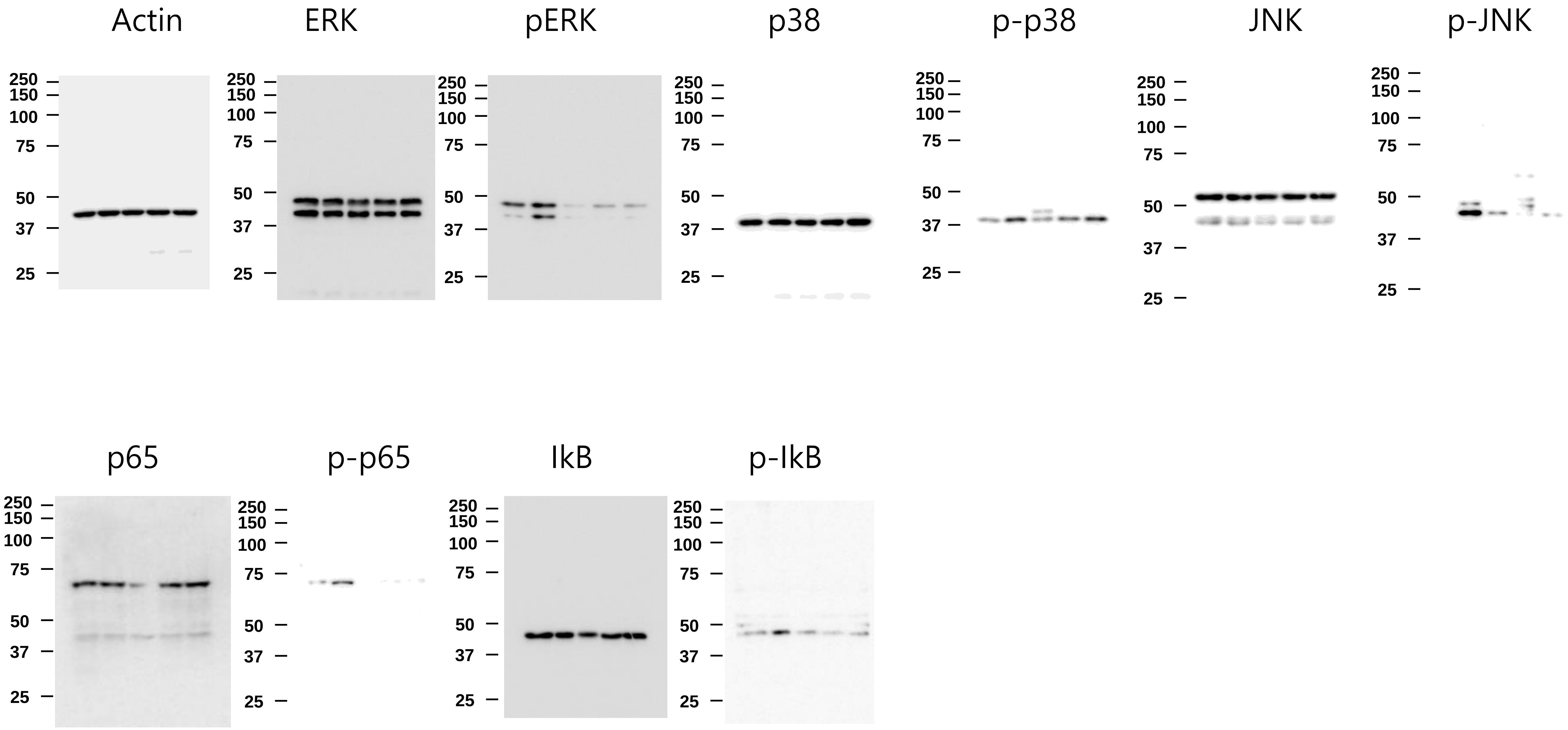

PM10 induced lung inflammation_lung tissue WB_3

ERK mem -> Actin

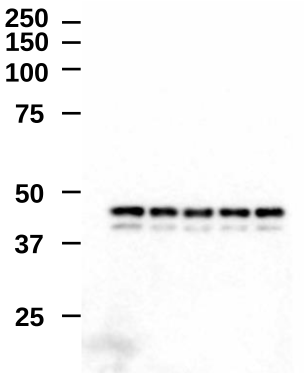

pERK mem -> Actin

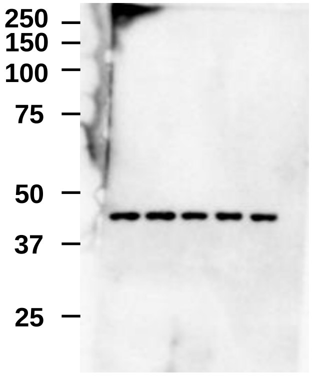

p38 mem -> Actin

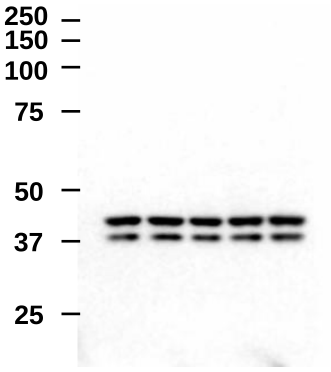

p-p38 mem -> Actin

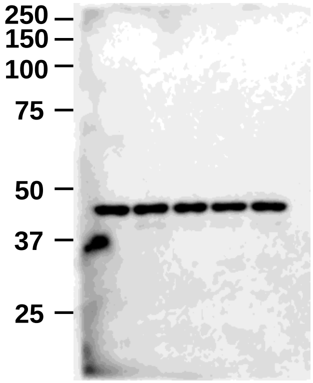

JNK mem -> Actin

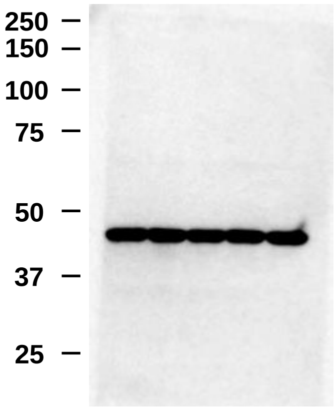

p-JNK mem -> Actin

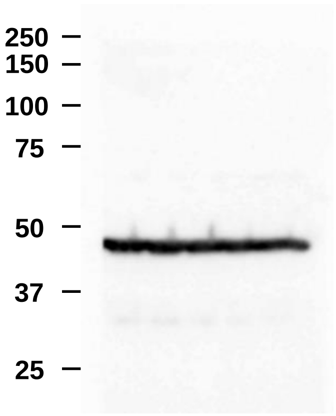

p65 mem -> Actin

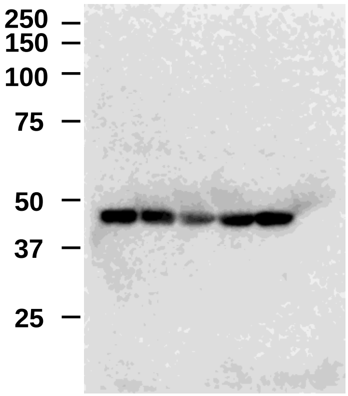

p-p65 mem -> Actin

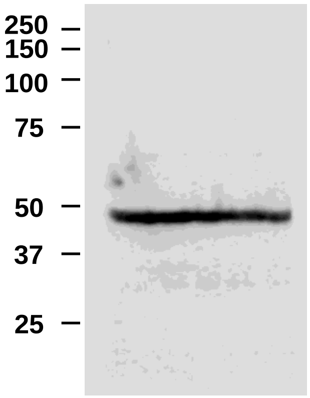

IkB mem -> Actin

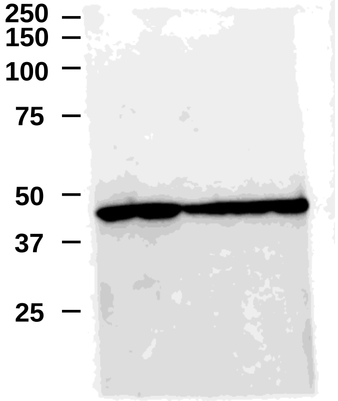

pIkB mem -> Actin

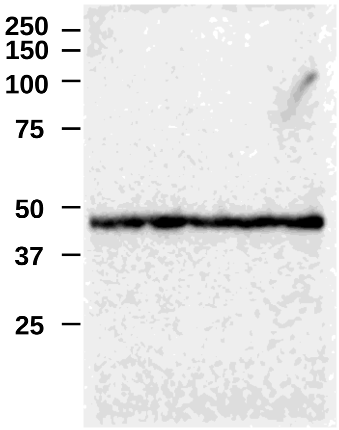

Supplement: Supplementary file 1 [file nutrients-15-04140-s001.zip › nutrients-2549336-supplementary.pdf]
